# HEALTH EFFECTS OF EXTENDED WORKING LIFE AMONG OLDER ADULTS

**DOI:** 10.1093/geroni/igae098.4315

**Published:** 2024-12-31

**Authors:** Rongfang Zhan, Elias Mpofu

**Affiliations:** University of North Texas, Denton, Texas, United States; University of North Texas, Denton, Texas, United States

## Abstract

**Objectives:**

To examine health benefits of working beyond the age of 66 in addition to the impact of socioeconomic status (SES) on potential health effects.

**Methods:**

This longitudinal study utilized data from 4,492 respondents, aged 50 years and over, from 6 waves (2004 - 2014) of the Health and Retirement Study. Measures included physical activities of daily living, mental health and the prevalence of chronic diseases in older adults.

**Results:**

Remaining in the workforce was associated with a reduced risk of mental illness. Socioeconomic disparities were apparent: extended working life had a beneficial effect on physical health and independence of older adults with lower SES and enhanced mental wellbeing of those with higher SES. No significant group differences in prevalence of chronic conditions was noted.

**Discussion:**

The current findings provide support for public policies aimed at increasing retirement age and have implications for how best to protect older worker’s health. Keywords: Older workers, retirement, socioeconomic status, health.

